# A review of plant-based coagulants for turbidity and cyanobacteria blooms removal

**DOI:** 10.1007/s11356-022-20036-0

**Published:** 2022-04-06

**Authors:** Widad El Bouaidi, Giovanni Libralato, Mountasser Douma, Abdelaziz Ounas, Abdelrani Yaacoubi, Giusy Lofrano, Luisa Albarano, Marco Guida, Mohammed Loudiki

**Affiliations:** 1 Laboratory of Water, Biodiversity and Climate Change; Phycology, Biotechnology and Environmental Toxicology Research Unit, Faculty of Sciences Semlalia, Department of Biology, Cadi Ayyad University, Av. Prince My Abdellah, P. O Box 2390, 40000 Marrakesh, Morocco; 2 Department of Biology, University of Naples Federico II, Complesso Universitario Di Monte Sant’Angelo, Via Cinthia 21, 80126 Naples, Italy; 3 Polydisciplinary Faculty of Khouribga (FPK), Sultan Moulay Slimane University, 25000 Khouribga, Morocco; 4 Laboratory of Applied Organic Chemistry, Faculty of Sciences Semlalia, Department of Chemistry, Cadi Ayyad University, 40000 Marrakesh, Morocco; 5 Dipartimento Di Scienze Motorie, Umane E Della Salute, Università Degli Studi Di Roma Foro Italico, Piazza Lauro De Bosis, 15, 00135 Roma, Italy

**Keywords:** Drinking water treatment, CyanoHABs, Turbidity, Coagulation/flocculation, Natural coagulants, Chemical coagulants

## Abstract

In recent years, the proliferation of Harmful Cyanobacterial Blooms (CyanoHABs) has increased with water eutrophication and climate change, impairing human health and the environment in relation to water supply. In drinking water treatment plants (DWTPs), the bio-coagulation based on natural coagulants has been studied as an eco-friendly alternative technology to conventional coagulants for both turbidity and CyanoHABs removal. Plant-based coagulants have demonstrated their coagulation efficiency in turbidity removal, as reported in several papers but its ability in cyanobacterial removal is still limited. This paper mainly reviewed the application of plant-based coagulants in DWTPs, with focus on turbidity removal, including cyanobacterial cells. The future potential uses of these green coagulants to reduce noxious effects of cyanobacterial proliferation are presented. Green coagulants advantages and limitations in DWTPs are reviewed and discussed summarizing more than 10 years of knowledge.

## Introduction

Harmful Cyanobacterial Blooms (CyanoHABs) in water supply systems become a worrying problem for water utilities worldwide (Anderson et al. 2002; Lopez et al., 2008). The frequency of occurrence of cyanobacteria blooms is mainly related to the increase in nutrient inputs produced by the anthropogenic activities and global warming (Chapra et al., 2017; Heisler et al., 2008; O’Neil et al., 2012; Paerl et al., 2011; Paerl and Huisman, 2009; Rigosi et al., 2015). Moreover, the combination of several environmental factors such as water temperature, thermic stratification, salinity, light intensity, stagnation and residence time, and nutrients concentration increase can promote cyanobacteria proliferation (Merel et al., 2013).

Various studies reported the presence of toxic cyanobacterial blooms in surface waters and at the intakes of drinking water treatment plants (DWTPs) (Carmichael et al., 2001; Lahti et al., 2001; McQuaid et al., 2011; Merel et al., 2010; Svrcek and Smith, 2004). They can cause several problems in DWTPs like increasing turbidity of water (Scheffer et al., 1993) and plugging of filters, reducing filter run-times and consequently increasing the backwash frequencies (Ho et al., 2012). In addition, cyanobacteria can produce toxic secondary metabolites called cyanotoxins including neurotoxins (homoanatoxin-a, anatoxin-a, anatoxin-a (S), homoanatoxin-a and saxitoxins), hepatotoxins (cylindrospermopsin, microcystins and nodularin), cytotoxins (debromoaplysiatoxin, lipopolysaccharide endotoxin, aplysiatoxin and lyngbyatoxin) and other compounds with adverse effects on skin, carcinogenic potentiality and ability to irritate the gastrointestinal tract (Briand et al., 2004; Brooks et al., 2016; Falconer and Humpage, 2005; Graham et al., 2010; Hitzfeld et al., 2000; Ho et al., 2012; Kaebernick and Neilan, 2001; Rastogi et al., 2015; Tokodi et al., 2018). Therefore, CyanoHABs constitute a a one-health treat (Christoffersen, 1996; De Figueiredo et al., 2004; Douma et al., 2017; Ghadouani and Coggins, 2011) potentially affecting all water sources (Carmichael et al., 2001; Codd et al., 2005; Smith and Schindler, 2009) with a relevant economic damage (Dodds et al., 2009; Steffensen, 2008). For this reason, the protection of water supplies from CyanoHABs becomes one of the major challenging priorities of the near future.

Several treatment methods including conventional processes (Zamyadi et al., 2013) and more recently, the utilization of ultrasonic irradiation, ultra-violet (UV) irradiation, hydrodynamic cavitation, electrocoagulation–flotation processes and chemical oxidants such as chlorine, potassium permanganate and ozone (Qi et al., 2021) are used in DWTPs to sanitize water from cyanobacteria and their deleterious effects (Meglič et al., 2017). However, cyanobacteria cells and cyanotoxins, which can cause cyanobacteria cells to grow in sand filters and enter the successive treatment systems or even the distribution network (Joh et al., 2011; Shekhar et al., 2017), are not easily removed by all water treatment technologies (Tang et al., 2017). Moreover, the application of these strategies can be constrained by the high investments required.

Coagulation/flocculation (C/F) based on the addition of chemical coagulants and flocculants, such as aluminium salts and ferric chloride (Betatache et al., 2014; Chen et al., 2013), which promotes the agglomeration of particles, known as flocs (Ghosh et al., 1994; Shin et al., 2008). It is considered the most important process for the removal of cyanobacterial cells in conventional DWTPs. It has been reported up to 90% of intracellular cyanotoxins removal (Ma et al., 2016; Sillanpää et al., 2018; Xagoraraki, 2007). Despite their efficiency in reducing turbidity and cyanobacterial cells, chemical coagulants and flocculants are still presenting detrimental effects, such as environmental pollution due to improper disposal of sludge, human health effects linked with the presence of residual alum in treated waters (Saleem and Bachmann, 2019; Simate et al., 2012), costly practices of sludge disposal and impacts on health and environment (Abdullah et al., 2016).

These drawbacks have motivated the search for natural coagulants and flocculants which are generally claimed to be more environmentally friendly in terms of production and usage to clean water from turbidity in water treatment plants. Natural coagulants can be ranked considering their origin and divided into three categories: i) plant-based coagulants; ii) animal-based coagulants; and iii) microorganism-based coagulants (Verma et al., 2012a). Among them, plant-based coagulants seem quite promising due to their available sources and relatively high performances gaining importance over the years (Choy et al., 2015). The main compounds responsible for green coagulation are polysaccharides (Kebaili et al., 2018; Kurane and Nohata, 1991; Miller et al., 2008; Prasertsan et al., 2006; Shamsnejati et al., 2015; Suh et al., 1997; Toeda and Kurane, 1991), poly-phenolic substances (Graham et al., 2010; M. Özacar, 2002; Sánchez-Martín et al., 20–10), functional proteins (Gassenschmidt et al., 1995; Ghebremichael et al., 2006; Ndabigengesere, KS. Narasiah, 1995), glycoproteins (Ferreira et al., 2011; Santos et al., 2009) and/or proteolytic enzymes (Horne et al., 2004). Natural coagulants and flocculants are advantageous thanks to their biodegradability, cost-effectiveness, safety and low amount of produced sludge than conventional ones (Asrafuzzaman et al., 2011; Bratby, 2006; Kumar and Quaff, 2018; Ndabigengesere, KS. Narasiah, 1995; Saleem and Bachmann, 2019; Swati and Govindan, 2005). Most studies focused on the effectiveness in turbidity removal, while only few ones considered the elimination of CyanoHABs.

The aim of this review paper is to summarize and discuss: i) plant-based coagulants and their use in water supplies turbidity treatment, evidencing ii) the missing information to research on to harness their potential to address the problem of CyanoHABs as an alternative to conventional coagulants and flocculants, as well as iii) the applicability and future challenges in DWTPs.

## Plant-based coagulants highlights

Recently, plant-based coagulants processes have become a major challenge for several scientists because of their efficiency, environmentally friendly behaviour compared to conventional coagulants, potential abundance and low cost (Antov et al., 2012; Betatache et al., 2014; Choy et al., 2016; Gautam et al., 2014; Šćiban et al., 2009).

Plant-based coagulants are made of polysaccharides or natural polymers, which are biodegradable, safe, easily available and easily accessible from reproducible agricultural resources (Asrafuzzaman et al., 2011; Bolto and Gregory, 2007; Bratby, 2006; Swati and Govindan, 2005). For instance, crude plant extracts are often available locally and can therefore be an inexpensive alternative to conventional coagulants. Moringa oleifera seeds is among the typical natural plant-based coagulants that is widely studied due to its efficiency performance to treat turbid water (Vunain et al., 2019). The plants such as rice starch and M. oleifera can be grown locally (Rasool et al., 2016; Ribau Teixeira et al., 2017). These reflections of producing coagulants could also strengthen societal aspect local populations depending on agricultural economics (Mahiya et al., 2016; Pondja Jr et al., 2017; Yin, 2010), guaranteeing the continuous supply of raw materials in the development of green coagulants (Mohd-Salleh et al., 2019).

Plant-based coagulants could also be obtained from agricultural wastes that are generally considered as not economically valuable having marketing constraints (Bories et al., 2009). Sutherland et al. (1994) stated that Moringa seeds are not only an oil source (40% wet weight of oil content), but after oil extraction, they can be reused as coagulants. Similarly, the derivatives from cassava (Manihot spp.) processing contain active coagulant agents (Heuzé et al., 2016; Howeler, 2001), based on natural polymers composed of proteins and polysaccharides (Mohd-Asharuddin et al., 2017; Zayadi et al., 2016). Several species from the Fabaceae family showed to contain chemicals that can be economically harvested to produce coagulants (Doyle 1994).

Plant-based coagulants are non-corrosive (Swati and Govindan, 2005) eliminating the risk of pipe erosions, and potentially carbon–neutral during their production process (Choy et al., 2014; Saleem and Bachmann, 2019). Moreover, they do not consume alkalinity, unlike chemical coagulants, and pH adjustments can be omitted (Choy et al., 2014). Operating costs in the water treatment sector are a great concern and plant-based coagulants seem an promising option (Kurita, 2006). Currently, Moringa cultivation costs approximately 2 USD /1 kg (i.e., ~ 3400 seeds), while 1 USD/1 kg is the alum salts quotation. Several efforts are necessary to really understand the life cycle impact analysis of these products and the role of the relative potential economy of scale, with particular reference to the social implications in local rural communities (Çoruh, 2005). Few data are available about the cost of raw coagulants as summarized in Table 1 where traditional and natural coagulants are compared (Çoruh, 2005). Bixler and Porse (2011) reported the unit price of some natural raw materials that reached the commercialization stage compared to chemical coagulants (Table 1), suggesting that chemical materials can be more expensive than some natural raw materials. Even if some chemical coagulants can be less expensive, several factors can contribute to increase the management cost like the necessity to adjust the water pH. For example, alginate is more expensive than alum salts, but it is used in smaller amounts making it cheaper than alum salts (Bixler and Porse, 2011; Çorhu, 2005). (Çorhu, 2005) evidenced that cost values are calculated considering only the chemical costs and not the operational costs associated with further treatment of sludge from the coagulation/flocculation process, that could further increase the whole cost associated to the use of traditional coagulants. For example, it has been reported that alum sludge disposal requires ~ 130 USD/ton (Maidon et al., 2015). (Ndabigengesere and Subba Narasiah, 1998) showed that under the same dosing conditions (1 mL/L) M. oleifera seeds generated a sludge volume of 1.5 mL/L compared to the 7.5 mL/L produced by alum salts. Plant-based coagulants can generate an amount of sludge that is approximately five times lower than chemical coagulants (Ndabigengesere et al., 1995) presenting suitable characteristics for agriculture reuse (Choy et al., 2014), with any further secondary pollution (Asrafuzzaman et al., 2011; Bolto and Gregory, 2007; Bratby, 2006; Swati and Govindan, 2005). The produced sludge is biodegradable and can be effectively degraded via biological methods (Renault et al., 2009). They can be disposed safely as soil fertilizer being not toxic (Gutierrez et al., 1998; Kaggwa et al., 2001; Mortula et al., 2009; Muisa et al., 2011; Verma et al., 2012b) thus reducing the cost of sludge management (Choy et al., 2014).

**Table 1:** Unit cost of natural raw materials and chemical substances and some raw materials in the commercialization phase; USD = US dollar; NTU = Nephelometric Turbidity Unit)

	Chemical substances		Natural raw materials		
	Alum sulphate	Polyelectrolyte	Calcium chloride	Alginate	Chitosan
Unit price (USD/kg)	0.042	0.23	0.081	0.29	-
Amount required (g/m^3^)	0.03	10^–4^	0.12	4 10^–4^	-
Total Price (USD/m^3^)	1.2 10^–6^	0.02 10^–6^	1 10^–4^	1 × 10^–7^	-
Initial turbidity in water (NTU)	5–100		80		-
Raw material used as coagulant on the commercialization scale					
Unit price (USD/kg)	0.3–0.5*	-	1	12	19
Amount used (mg/L)	3	-	80	0.2	-

^*^Prices obtained from the bulk suppliers in Turkey (Bixler and Porse, 2011)

## Potentialities of plant-based coagulants to remove turbidity from water

The effectiveness of plant-based coagulants was investigated by several authors to treat water contaminated by toxic cyanobacteria, excess of turbidity, heavy metals (Mahiya et al., 2016), including wastewater as well (Yin, 2010; Choy et al., 2015; Oladoja and Pan, 2015; Villaseñor-Basulto et al., 2018; Mohd-Salleh et al., 2019; Saleem and Bachmann, 2019). The application of plant-based coagulants for turbidity removal is summarized in Table 2 considering water test sample of water, type of extraction, contact time, initial turbidity and removal efficiency, temperature (°C), optimum coagulant dose (g/L) and pH. The average turbidity removal rates were approximately of 86% with abatement rates up to 95% like for *Trigonella foenumgraecum* and *Abelmoschus esculentus* at pH 3.17–3.20 (Khoo et al., 2021; Mohammad Lanan et al., 2020), and up to 77% for *Tacca leontopetaloides* at pH 3 (Makhtar et al., 2021), even though the considered pH values made water not immediately suitable for human consumption (WHO, 2007). Conversely, *M. oleifera* removed turbidity up to 97% at pH 6.8 (15 g/L) and up to 86–94% at pH 6.2 (0.15 mg/L) (Nhut et al., 2020; Vunain et al., 2019). Seeds of *M. oleifera* have been used as efficient natural coagulants in certain developing countries unaffordable for conventional chemicals (Bhatia et al., 2007). Its seeds can contain up to 34%, 15% and 16% of proteins, carbohydrates and lipids, in that order. (Othmani et al., 2020) suggested that the high amount of protein can be responsible of its great activity as coagulant. Cactus evidenced the ability to reduce turbidity up to 92% and 98% with 0.5 g/L at pH = 8.89 and pH = 6, respectively (Wan et al., 2019; Choudhary et al., 2019b). The reduction of pH between 7.00 and 8.00 showed a turbidity removal efficiency up to 98% with 1.5 g/L of cactus-based coagulant. Cactus mainly contains water (80–95%), fibre (1–2%), proteins (0.5–1%) and carbohydrates (3–7%) (Ginestra et al., 2009), and are recognized for the presence of mucilage, that is assumed to be responsible for the coagulation/flocculation activity.

**Table 2:** List of plant material investigated as natural coagulants to remove turbidity. RT = room temperature, n.a. = not available

Natural coagulant	Test sample of water	Type of extraction	Contact time	Initial turbidity	Temperature (°C)	Optimum coagulant dose (g/L)	Optimum pH	Turbidity removal efficiency (%)	References
*Abelmoschus esculentus*	Palm oil mill effluent	Fresh Okra	Fast stirring for 2 min Slow stirring of 60 rpm for 30 min	7700—13,600	n.a	116	3.2	94.97	(Khoo et al., 2021)
*Trigonella foenumgraecum*		Fenugreek seeds				4.1			
*Trigonella foenum-graecum*	Palm oil mill effluent	Fenugreek seeds	Fast stirring for 2 min Slow stirring of 60 rpm for 30 min	7700—22,200	n.a	0.409	3.17	94.97	(Mohammad Lanan et al., 2020)
*Abelmoschus escluentus*		Fresh Okra				5.77			
*Citrus aurantiifolia* and *Citrus microcarpa*	Synthetic water using kaolin	Peel extract	Fast stirring of 120 rpm for 3 min Slow stirring of 50 rpm for 20 min	115.83	n.a	0.05	6.5	77.60	(Dollah et al., 2020)
*Tacca leontopetaloides*	Raw leachate	*T. leontopetaloides tuber*	Fast stirring of 200 rpm for 4 min Slow stirring of 40 rpm for 30 min	218	RT	0.24	3	76.99	(Makhtar et al., 2021)
*M. oleifera*	Wastewater treatment plant	*M. oleifera* seeds	-	287 ± 3	22.4	15	6.8	97.30	(Vunain et al., 2019)
	Surface water	*M. oleifera* seeds	Fast stirring of 120 rpm for 2 min Slow stirring of 50 rpm for 30 min	n.a	27 ± 0.5	0.15	6.2	Rainy season 87.8 to 93.3 Dry season 85.7 to 94.3	(Nhut et al., 2020)
	Raw water	*M. oleifera* seeds	Initial stirring of 50 rpm for 15 min Fast stirring of 150 rpm for 10 min Slow stirring of 50 rpm for 15 min	15.6 ± 0.64	RT	1.64	n.a	82.04	(Pandey et al., 2020)
*M. oleifera*	River water Pond water Ground water	*M. oleifera* seeds	n.a	Stream: 20.5 Pond: 125 Well: 10.7	n.a	4.5 6 2.6	6.8 5.3 7	95.56 66.96 90.37	(Egbuikwem and Sangodoyin, 2013)
*M. oleifera*	River water	*M. oleifera* pods	Fast stirring of 200 rpm for 2 min Slow stirring of 40 rpm for 5 min	632 ± 3.20	n.a	12	7.45	99.20	(Jodi et al., 2012)
*M. oleifera*	Ground water	*M. oleifera* seeds	Stirring for 45 min at 110–120 rpm	12.4 ± 0.02	n.a	15	8	75	(Mangale Sapana et al., 2012)
*M. oleifera*	River water	*M. oleifera* seeds	Between 5 and 60 min	123.3	n.a	1.6	7.5	> 85	(Sánchez-Martín et al., 2012)
*M. oleifera*	Pond water	*M. oleifera* seeds	n.a	130.1	27	n.a	7.6	76.36	(Yongabi et al., 2011)
*M. oleifera*	River water	*M. oleifera* seeds	n.a	n.a	10	5	7.5	71.02	(Beltrán-Heredia and Sánchez-Martín, 2009)
*M. oleifera*	Tap water (kaolin)	*M. oleifera* seeds	n.a	105	20 ± 1	1	7.6	93.33	(Ndabigengesere and Subba Narasiah, 1998)
*Cassava* peels	Wastewater treatment plant	Cassava peels starch	n.a	194 ± 14.43	n.a	44.8	6	60.19	(Kumar et al., 2020)
*Quercus branti*	Synthetic turbid water using kaolin suspension	Oak fruit	Fast stirring of 200 rpm for 1 min Slow stirring of 70 rpm for 30 min	20 to 250	RT	6.22	n.a	63.5	(Jamshidi et al., 2020)
Pistachio green	Synthetic turbid water using kaolinite	Pistachio green hull	n.a	300	n.a	5	5	88	(Nasrabadi et al., 2020)
*Guazuma ulmifoliya*	Synthetic dairy wastewater	*G. ulmifolia* stem barks	Fast stirring of 200 rpm for 1 min Slow stirring of 30 rpm for 15 min	698 ± 9.4	n.a	77.58	5	95.8	(Muniz et al., 2020)
*Cactus*	Wastewater treatment plant	Cactus pads	Fast stirring of 160 rpm for 5 min Slow stirring of 40 rpm for 25 min	50	17 ± 1	2.8	12	98.33	(Ayat et al., 2021)
*Cactus opuntia (ficus-indica)*	Tailings pond water	Cactus mucilage	n.a	80 ± 2	23 ± 1	0.5	6	98	(Wan et al., 2019)
						0.5	8.89	91.49	
*Cactus opuntia (ficus-indica)*	Stimulated industrial water-based paint wastewater	Eluted on 3 N NaCl	n.a	n.a	n.a	n.a	n.a	78.43	Vishali and Karthikeyan (2015b)
						30	n.a	80.44	
						n.a	5	99.67	
						100	n.a	94	
*Opuntia ficus-indica*	Oil sands process-affected water	Cladodes of *Opuntia ficus indica*	n.a	n.a	n.a	1.5	7–8	98	(Choudhary et al., 2019b)
Pine cone	Synthetic turbid water	Pine cones	n.a	67, 69, 71 and 75	n.a	0.5	2	77	(Hussain et al., 2019)
							12	76	
*Cicer arietinum*	Palm oil mill effluent	Chickppea seeds	n.a	17,600	n.a	2.6	6.69	86	(Choong Lek et al., 2018)
*Flower of Musa sp.*	Effluents from the processing of iron ore	Extraction of tannins	Fast stirring for 2 min Slow stirring for 15 min	86,500	n.a	8.5	6.25	97.58	(Vaz et al., 2018)
*Maerua decumbent*	Paint industry wastewater	*M. decumbent* roots	Fast stirring of 180 rpm for 3 min Slow stirring of 20 rpm for 30 min	2575	20 ± 2	1	5.56	99.24	(Kakoi et al., 2017)
Corn and potato	Synthetic turbid water using kaolin suspension	Conventional starches	Fast stirring of 100 rpm for 2 min Slow stirring of 40 rpm for 20 min	165 ± 5	25 ± 1	0.12	4	50	Choy et al. (2016)
*Plantago ovata*	Raw surface water	Plantago seeds extracted by using FeCl3-induced extract	Fast stirring of 120 rpm for 1 min Slow stirring of 45 rpm for 10 min	76	24	0.025	< 8	95.6	Ramavandi (2014)

## Potentiality of plant-based coagulants to remove cyanobacteria

Currently, few studies have been reported to mitigate CyanoHABs with plant-based coagulants as summarized in Table3. Cyanobacterial removal was ≥ 70% for all the plant-based coagulants. According to El Bouaidi et al. (2020), *Vicia faba* seeds and *Opuntia ficus indica* cladodes removed up to 85% of *M. aeruginosa* from treated water using 0.5 and 1 g/L (pH 5) of the relative coagulants, respectively. Teixeira et al. (2017) evaluated the potential of *M. oleifera* to remove *M. aeruginosa* from water using a process including coagulation, flocculation and dissolved air flotation (DAF). Results demonstrated that this plant-based coagulant can remove ~ 80% of *M. aeruginosa* cells. Camacho et al. (2015) explored the potential effect of *M. oleifera* at low turbidity level to sanitize water contaminated by cyanobacteria evidencing its ability to reduce chlorophyll-a and turbidity up to 60%, as well as suspended organic matter (40–50%).

**Table 3:** List of plant materials used as natural coagulant to mitigate cyanobacteria. RT = room temperature, n.a. = not available, n.e. = not effect

Plant-based coagulants	Extract type	Test sample of water	Target cyanobacteria	Contact time	Initial turbidity	Temperature (°C)	Optimum coagulant dose (g/L)	pH	removal efficiency (%)	Reference
*Vicia faba* and *Opuntia ficus indica*	Faba been seeds and cactus cladodes	Synthetic water prepared from cyanobacterial cells density of 10^6^ cells/mL	*Microsystis aeurigonsa*	Fast stirring of 200 rpm for 2 min Slow stirring of 40 rpm for 30 min	200	RT	0.5 and 0.1	5	Cyanobacteria cells: > 85	(El Bouaidi et al., 2020)
*Opuntia strica* Haw	Cactus cladodes	Surface water from Bodocongó reservoir	Cyanobacterial bloom containing: *Microcystis aeruginosa, Sphaerocavum Brasiliense*, *Cylindrospermopsis racibroskii* (Woloszynska) and *Plankthotrix isothrix* (Skuja)	5, 15, 30, 60 and 120 min	58.1 ± 1.5	25	10	n.e	Turbidity: 52 Cyanobacteria cells: 70	(Nery et al., 2019)
*Moringa olifera*	MO seeds proteins (albumin and globulin)	Distilled water contaminated with cyanobacterial cell of density of 10^4^ cells/mL	*Microcystis aeruginosa*	Coagulation at a velocity gradient of 315 s^−1^ for 20 s; flocculation at a velocity gradient of 15 s^−1^ for 10 min	34.7 ± 0.61	n.a	10	7.77	Cyanobacterial cells: 83.87	(de Oliveira Ruiz Moreti et al., 2019)
Pomegranate peel	Pomegranate peel tannins	Synthetic water prepared from cyanobacterial cells density of 10^6^ cells/mL	*Microcytis aeruginosa*	n.a	n.a	n.a	0.2	7.4	Cyanobacteria cells: 94.22	(Wang et al., 2018)
*Moringa oleifera*	MO seeds	Synthetic surface water using cyanobacterial cells	*Microcytis aeruginosa*	Coagulation for 2 min with a velocity gradient of 380 s^−1^ (200 rpm), flocculation for 8 min at 70 s^−1^ (20 rpm)	n.a	21.0 ± 1.0	5	7.4	80	(Teixeira et al., 2017)
*Moringa oleifera*	MO seeds extracts by 1 M NaCl and CaCl_2_	Synthetic water spiked with humic acid and cyanobacteria cells	*Microcytis aeruginosa*	Coagulation at a velocity gradient of 1000 s1 for 10 s^−1^, flocculation at a velocity gradient of 15 s^−1^ for 15 min	25.3 ± 0.3	25 ± 2	5	8.3	Cyanobacteria cells: 79.9	(Carvalho et al., 2016)
*Moringa oleifera*	MO seeds	Artificially water contaminated with cyanobacteria (order of 10^4^ cells/mL)	*Anabaena flos-aquae*	Rapid mixing gradient of 315 and 850 s^−1^ for 20 S Slow mixing gradient of 5, 10 and 15 s^−1^ for 10, 15 and 20 min	30 ± 0.5	25 ± 2	10	7.0–7.7	Cyanobacteria cells: 96.4	(Moreti et al., 2016)
*Moringa oleifera*	MO seeds Saline extraction KCl and NaCl (1 M)	Deionized water with an inoculum of cyanobacteria cells Turbidity ranging from 50–450 NTU	*Microcystis protocystis*	8 min of retention time	between 50 and 450	25 ± 2	5	7.32	Cyanobacteria cells: between 80 and 95	(Camacho et al., 2015)

Thus, the removal of cyanobacteria and cyanotoxins in DWTPs can be carried out through two groups of methods: i) effective in removing intracellular cyanotoxin with intact cyanobacterial cells, and ii) eliminating extracellular cyanotoxin removing organic matter (Xagoraraki, 2007). To increase the whole performance of water treatment, reactions can occur sequentially in two separated reactors (Gitis and Hankins, 2018). Several treatment techniques are used in order to increase the performance of water treatment methods, *e.g.* photolysis with UV radiation at 254 and 185 nm (Chintalapati and Mohseni, 2020), adsorption process with activated carbons (Pendleton et al., 2001; Zhang et al., 2011) and hydrophyte filter bed (Song et al., 2009). Coagulation/flocculation has been widely applied in combination with ultrafiltration, as an effective pre-treatment, to improve the removal of natural organic matter and to reduce membrane fouling (Liu et al., 2017). There is a great need to further research on the coagulation/flocculation process to identify the best practice to reduce effects of CyanoHABs considering also low tech-content methods.

## A critical view on the applicability and future challenges of plant-based coagulants

(Sillanpää et al., 2018) evidenced that the use of plant-based coagulants for the removal of suspended particles and natural organic matter in WWTPs is still underexplored. Currently, most results are laboratory based focusing on controlling water turbidity by studying the mechanism of these natural coagulants through charge interaction and bridging mechanism that is attributed to the pair nature of the treated water and plant-based coagulant tested. According to (Ang and Mohammad, 2020), natural coagulants can record poor removal performance when the treated water contains many constituents such as suspended solids, heavy metals and microalgae, thus requiring several combined processes in order to meet the expected goal.

Several studies (Choudhary et al., 2019a; Vunain et al., 2019; Wan et al., 2019) highlighted that plant-based coagulants have been used in various types of effluents saturated with different suspended solids ranging from wastewater, water from paint factories and artificially turbid water. The originality of the adoption of plant-based coagulants is related to the potential sources supplying the reagents like plant, including invasive species, or weeds (i.e., including seaweed), and plant waste. Some direct critical aspects in the use of plant-based coagulants are i) the lack of plants for mass processing; ii) the perception of a low-volume market; and iii) the lack of regulations stipulating the quality of processed coagulant extracts (Sutherland et al., 2002); iv) storage can be affected by microbial degradation causing undesired loss of reagents (Albaliwano et al., 2003; SAMIA et al., 1979).

In term of commercialization, few natural coagulants have reached the market, although several various native plant extracts have been identified as suitable for coagulation activities in removing turbidity and cyanobacteria (Sowmeyan et al., 2011). Currently, only M. oleifera seeds extracts are well documented with full-scale application in coagulation processes (Sutherland et al., 2002). Some critical points can affect the future employ of plant-based coagulants like the regular supply of raw materials mainly due to the relative economy of scale. For example, M. oleifera seeds can be harvested twice a year (Radovich, 2009) and there are still no clear estimate if the expected production will satisfy the possibility of a whole replacement of traditional chemical coagulants, or can represent just an integration and/or a partial substitution, greening just part of the process.

Another potential drawback identified from plant-based coagulants is the increase of organic load in the treated mass of water, as chemical oxygen demand (COD) and biological oxygen demand (BOD) (Sánchez-Martín et al., 2012), that can further promote microbial growth and potentially increase the frequency in clogging at the filtration stage of DWTPs. As a result, increased COD level can be a disadvantage if treated water is stored for a long period of time or requires chlorination (Sánchez-Martín et al., 2012). Distilled water extracts from M. oleifera (1%) can contain approximately 88.8 g/L of COD (Baptista et al., 2015), while the saline extraction up to 175 g/L.

## Conclusions

Plant-based coagulants in DWTPs are an interesting and promising approach for the water sector that must be attentively evaluated, especially to integrate traditional chemical reagents. For sure, they cannot be considered as an overnight solution, but a medium-term potential option for greening the processes of coagulation/flocculation and cyanobacterial bloom removal. Several flaws are currently present and are mainly associated not only to the lack of data about full-scale applications, but also to the potential increase in treated water COD, the limited availability of adequate plant biomass and its potential biodegradability during storage conditions. The main advantages are related to the ability to support coagulation/flocculation treatments with efficiencies quite like to traditional reagents including costs, that with the relative economy of scale, could be potentially further reduced. Future focused research activities must elucidate: i) suitable species in an agricultural production perspective; ii) cost–benefit analysis; and iii) full-scale potential applicability.

## Conflicts of interest

The authors declare no conflict of interest.

## Declarations

### Availability of data and materials

All data generated or analysed during this study are included in this published article.
